# Poly(3-hydroxybutyrate-*co*-3-hydroxyvalerate) Blends with Poly(caprolactone) and Poly(lactic acid): A Comparative Study

**DOI:** 10.3390/polym15234566

**Published:** 2023-11-29

**Authors:** Carmen R. Tubio, Xabier Valle, Estela Carvalho, Joana Moreira, Pedro Costa, Daniela M. Correia, Senentxu Lanceros-Mendez

**Affiliations:** 1 BCMaterials, Basque Center for Materials, Applications and Nanostructures, UPV/EHU Science Park, 48940 Leioa, Spain; xabiervalle97@gmail.com (X.V.); senentxu.lanceros@bcmaterials.net (S.L.-M.); 2 Physics Center of Minho and Porto Universities (CF-UM-UP) and LaPMET—Laboratory of Physics for Materials and Emergent Technologies, University of Minho, 4710-057 Braga, Portugal; estelacarvalho690@gmail.com (E.C.); joanacdias97@gmail.com (J.M.); pcosta@fisica.uminho.pt (P.C.); 3 Institute of Science and Innovation for Bio-Sustainability (IB-S), University of Minho, 4710-057 Braga, Portugal; 4 Centre of Chemistry, University of Minho, 4710-057 Braga, Portugal; dcorreia@quimica.uminho.pt; 5 IKERBASQUE, Basque Foundation for Science, 48009 Bilbao, Spain

**Keywords:** biopolymer blends, poly(hydroxybutyrate-*co*-hidroxyvalerate) (PHBV)

## Abstract

Poly(hydroxybutyrate-*co*-hidroxyvalerate) (PHBV) is a biodegradable polymer, which is a potential substitute for plastics made from fossil resources. Due to its practical interest in the field of tissue engineering, packaging, sensors, and electronic devices, the demand for PHBV with specific thermal, electrical, as well as mechanical requirements is growing. In order to improve these properties, we have developed PHBV blends with two thermoplastic biodegradable polyesters, including poly(caprolactone) (PCL) and poly(lactic acid) (PLA). We analysed the effect of these biopolymers on the morphological, wetting, structural, thermal, mechanical, and electrical characteristics of the materials. Further, the biodegradation of the samples in simulated body fluid conditions was evaluated, as well as the antibacterial activity. The results demonstrate that the blending with PCL and PLA leads to films with a dense morphology, increases the hydrophilic character, and induces a reinforcement of the mechanical characteristics with respect to pristine PHBV. In addition, a decrease in dielectric constant and a.c. electrical conductivity was noticed for PHBV/PLA and PHBV/PCL blends compared to neat PHBV polymer. All neat polymers and blends showed antibacterial properties against *S. aureus*, with more than 40% bacterial reduction, which increased to 72% in the presence of PCL polymer for a blend ratio of 50/50. Thus, it is demonstrated a suitable way to further tailor a variety of functionalities of PHBV for specific applications, by the development of polymer blends with PLA or PCL.

## 1. Introduction

Polyhydroxyalkanoates (PHAs) and copolymers comprise a family of natural biodegradable polyesters, which are produced from microorganisms [1,2,3]. These biodegradable, biocompatible, and thermoplastic biopolymers have received much attention because of their potential use in many areas, including tissue engineering [4], packaging [5], agriculture [6], and microfiltration membranes [7], among others. Interestingly, PHA is presented as a potential alternative to replace conventional petroleum-based plastics in a wide range of industrial applications [8]. However, it still needs to improve mechanical properties and thermal stability to achieve specific application requirements. Hence, production strategies are constantly investigated to tailor the mechanical and thermal properties of the PHA family to promote their use in a wide range of applications. Special emphasis is placed on poly(3-hydroxybutyrate-*co*-3- hydroxyvalerate) (PHBV) co-polymers due to their superior physicochemical properties, including thermal and mechanical, compared to other PHAs [9,10]. Also, PHBV is characterized by its piezoelectricity, resistance to ultraviolet radiation, and thermoplasticity [11,12]. However, PHBV copolymer has high crystallinity and brittleness, low impact resistance, and poor electrical properties, limiting its application. Various routes have been explored to improve these characteristics, including blending with other polymers, additives or fillers, functionalization by chemical grafting, and strategies for biological production, among others [9,13].

Blending has been reported as an effective way to improve and tailor the properties of PHBV to certain applications. Common polymers for this strategy include poly(lactic acid) (PLA) [14,15], poly(ethylene succinate) (PES) [16], poly(butylene-*co*-succinate-*co*-adipate) (PBSA) [17,18], poly(caprolactone) (PCL) [19,20], polyurethane (PU) [21,22], and cellulose [23,24]. Moreover, other functional fillers have been evaluated including graphene nanoplatelets [25] or carbon nanotubes [26] as conductive fillers, superparamagnetic iron oxide nanoparticles for biomedical applications [27], and silver as an antibacterial agent [28], among others. Optimization of PHBV-based blends depends on various factors, such as composition and control of morphology, which depend on processing techniques and post treatments. Therefore, to further exploit and optimize the performance of PHBV-based blends, it is necessary to establish proper composition–processing–functional characteristics relationships.

In this context, the novelty of the work relies on the systematic study of the processing, characterization, and evaluation of PHBV-based blends composed of PCL and PLA biopolymers in other to tailor their physical–chemical properties. These biopolymers were chosen because they are thermoplastic and biodegradable, environmentally friendly, as well as showing suitable mechanical properties [29,30,31,32]. PHBV-based blends with different composition ratios (100:0, 75:25, 50:50, 25:75, and 0:100) were prepared. The functional properties of these polymer blends were evaluated including morphology, wetting, structural, thermal, mechanical, electrical, degradability, and antibacterial activity characteristics. Correlation between these properties and blend ratio composition is essential for the development of PHBV-based blends with a targeted range of properties for specific applications, including sensing and tissue engineering, among others. Our work demonstrate new strategies for developing high-performance sustainable polymer-based applications with tailored characteristics and also provide an understanding of the influence of blend ratios on the characteristics of PHBV-based blends.

## 2. Materials and Methods

### 2.1. Materials

Poly(3-hydroxybutyrate-*co*-3-hydroxyvalerate) (PHBV), PHI 003 was supplied by Natureplast (Mondeville, France). PHI 003 is a PHBV copolyester with a density of 1.24 g·mL^−1^ and melt flow rate MFI of 15–30 g/10 min (tested at 190 °C and 2.16 kg). Poly(caprolactone) (PCL) with an average molecular weight of Mn = 80,000 was obtained from Sigma-Aldrich. Poly(lactic acid) (PLA) pellets (Ingeo biopolymer 3052D) with a density of 1.24 g·mL^−1^ and melt flow MFR of 14 g/10 min (tested at 210 °C and 2.16 kg) were purchased from Natureworks LCC, Minneapolis, MN, USA. Chloroform was obtained from Sigma-Aldrich, St. Louis, MI, USA. All reagents were used as received. 

### 2.2. Sample Preparation

Solution mixing of PHBV/PCL blends using chloroform as a solvent was prepared at different weight ratios of PHBV/PCL: 100/0, 75/25, 50/50, 25/75, 0/100. First, the neat polymer (PHBV and PCL) solutions were prepared by dissolving 1 g of the specific polymer in 6 mL of chloroform under constant stirring. After, the PHBV solution was mixed with the PCL solution, and they were magnetically stirred for 2 h to obtain a homogeneous mixture. The resultant solutions were cast onto a glass substrate by doctor blade, and the films were dried at ambient temperature to allow the complete evaporation of the solvent. The thickness of the films is in the range of 80 to 100 µm. PHBV/PLA samples were also prepared under the same processing procedure and conditions.

### 2.3. Characterization of the Samples

Polymer blend morphology was examined by scanning electron microscopy (SEM, Hitachi S-4800, Japan) at an accelerating voltage of 15 kV. Before analysis, cryogenically-fractured samples were coated with a gold layer by sputtering with a Polaron SC502 apparatus.

Water contact angle measurements were carried out by using a contact angle goniometer (OCA 15EC, Neurtek, Gipuzkoa, Spain). Three measurements were performed in each sample under static conditions and at room temperature.

The attenuated total reflectance Fourier transform infrared spectroscopy (ATR-FTIR) test was performed with Jasco FT/IR-4100 equipment (Easton, MD, USA). FTIR spectra were obtained from 4000 to 600 cm^−1^ with 64 scans and a resolution of 4 cm^−1^.

Thermal transitions were analysed by differential scanning calorimetry (DSC). DSC was carried into the PerkinElmer 8000 instrument (Waltham, MA, USA) under N2 flow in the temperature range between −70 and 200 °C at a rate of 10 °C min^−1^. 

The mechanical behaviour of samples was evaluated under tensile testing by using an Autograph AGS-J from Shimadzu (Kyoto, Japan) with a 50 N load cell. Stress–strain curves were obtained at a strain rate of 5 mm·min^−1^ for samples 5 mm in width and 35 mm in length.

Room temperature dielectric measurements were carried out by measuring the capacity (*C*) and the dielectric losses (tan *δ*) with a Quadtech 1920 Precision LCR Meter (USA) in the 20 Hz–1 MHz frequency range with an applied voltage of 0.5 V. The real (*ε′*) and imaginary (*ε″*) parts of the dielectric function were obtained according to [33]:

(1) $$\varepsilon^{\prime }=\frac{C\times d}{A}$$

(2) $$\varepsilon^{\prime \prime }=tan\delta \times \varepsilon^{\prime }$$

where *C* is the capacity, *d* is the thickness sample, *A* indicates the area, and tan δ is the loss tangent. The a.c. conductivity (*σ′*) was calculated from the dielectric measurements using:

(3) $$\sigma^{\prime }=\varepsilon_{0}\times w\times \varepsilon^{\prime \prime }$$

where *ε_0_* (8.85 × 10^−12^ F·m^−1^) is the permittivity of free space and w=2πf is the angular frequency.

### 2.4. Degradation Assays

For the degradation assays, simulated body fluid (SBF) was prepared. Briefly, SBF was obtained by adding to 700 mL of ultrapure water under mechanical agitation the following components: NaCl (8.04 g), NaHCO_3_ (0.36 g), KCl (0.23 g), K_2_HPO_3_H_2_O (0.23 g), MgCl_2_·6H_2_O (0.31 g), HCl (39 mL), CaCl_2_ (0.29 g), Na_2_SO_4_ (0.07 g), and TRIS (6.12 g). After dissolution, the pH was adjusted to 7.4 and completed the volume of 1 L. Samples from the different materials were cut into 1 cm × 1 cm pieces and immersed in 3 mL of SBF at 37 °C in well tissue culture polystyrene plates. To prevent pH and ion concentration variations, the fluid was changed once per week during the 8-week period. The degradation of the samples was evaluated by weight loss measurements and UV-Vis spectroscopy analysis.

For weight evaluation, all samples were weighed before the beginning of the assay and after 8 weeks. The samples were washed 3 times with ultrapure water, left to dry at room temperature for 5 days, and weighed using a Sartorius M5 microbalance with a readability of 0.001 mg. On the other hand, after the immersion, the SBF solutions were measured by UV-Vis absorption spectroscopy (Infinite M Nano, Tecan, Kawasaki, Japan) at the 230–600 nm range each week for 8 weeks.

### 2.5. Antibacterial Testing

The bacteriostatic activity of the developed materials was evaluated using the standard shake flask method (ASTM-E2149–01). Briefly, two different pre-inoculums were prepared using a single colony from a stock bacterium cultures of Gram-positive *Staphylococcus aureus* (*S. aureus*, ATCC 25923) and Gram-negative *Escherichia coli* (*E. coli*, ATCC 25922), acquired from the American Type Culture Collection (ATCC LGC Standards, Spain). Both pre-inoculums were incubated overnight at 37 °C and 110 rpm in nutrient broth (NB). After approximately 20 h, they were harvested twice by centrifugation at 5000 rpm for 5 min and then suspended in a buffer solution containing 0.9% NaCl (*w*/*v*). The optical density (OD) of the bacterial cultures was measured at 600 nm and adjusted for both bacteria resulting in a working inoculum of 1 × 107 CFU·mL^−1^. Small segments (1 cm × 1 cm) of the samples were sterilized with UV light and brought into contact with 1 mL of the bacterial suspension, incubated at 37 °C and 200 rpm for 2 h. Subsequently, to determine the number of surviving bacteria, the suspensions were diluted in a sterile buffer solution, plated on NB agar, and incubated at 37 °C for 24 h.

This method provides quantitative data that allows for calculating the rate of colony formation decrease, which is then converted to the average colony forming units per milliliter of the buffer solution in the flask (CFU·mL^−1^). According to Equation (4), the results are expressed in terms of bacterial growth inhibition.

(4) $$Bacterial\;growth\;inhibition\%=\frac{(A-B)\times 100}{A}$$

where *A* and *B* represent the average number of viable bacteria before and after contact with the samples, respectively. The data are shown as the mean values ± SD (*n* = 3). 

## 3. Results

### 3.1. Morphological Analysis and Wettability

**Figure 1:**
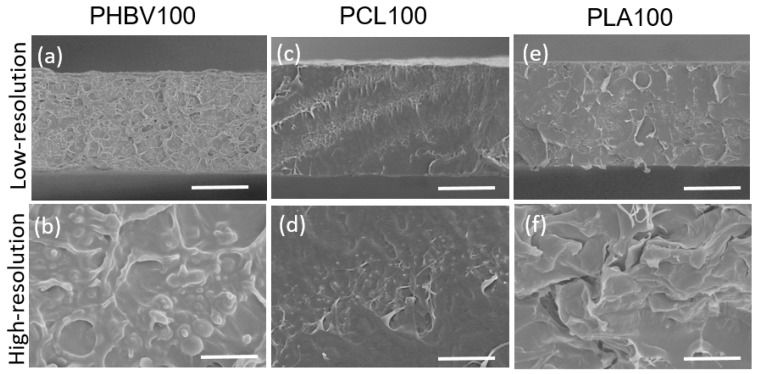
Cross-sectional SEM images of neat polymers (**a**,**b**) PHBV, (**c**,**d**) PCL, and (**e**,**f**) PLA, at low (scale bar: 30 µm) and high (scale bar: 5 µm) resolution.

**Figure 2:**
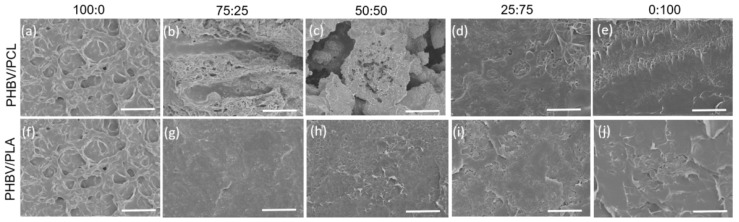
Cross-sectional SEM images of samples with different (**a**–**e**) PHBV/PCL ratios and (**f**–**j**) PHBV/PLA ratios. Scale bar 10 µm.

SEM images allow us to highlight the microstructure evolution of the PHBV-based blends with compositional variation. Figure 1 shows the cross-sectional SEM images of the neat polymers at different resolutions. Obvious differences are observed between polymers, where PHBV (Figure 1a,b) presents an open cell morphology, while PCL (Figure 1c,d) and PLA (Figure 1e,f) show a compact and dense structure. Furthermore, Figure 2 shows the cross-sectional SEM images of the different blends. When the concentration of PCL or PLA in the blend is increased, samples become more compact, showing a more dense morphology with no indications of phase separation. Similar results were previously reported in blends made with PCL [34]. From these observations, it can be concluded that blending with high contents of PCL and PLA mixed with PHBV forms homogeneous and dense microstructures, contributing to obtaining improved mechanical properties with respect to neat PHBV.

**Figure 3:**
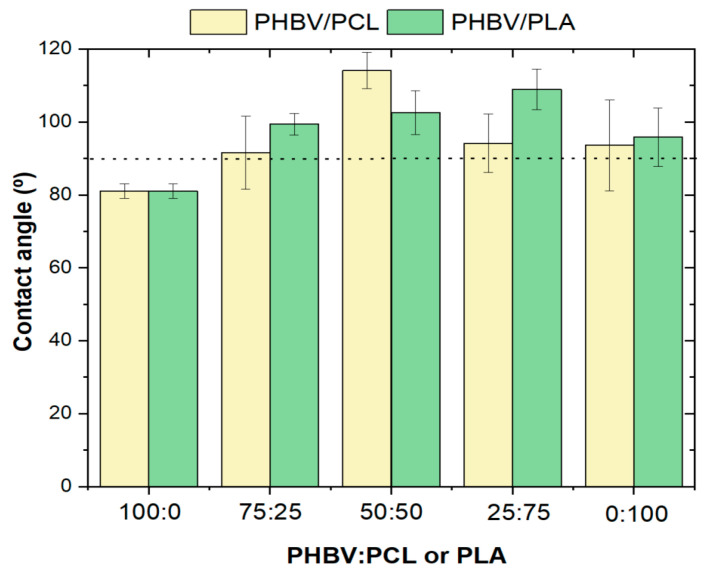
Contact angle values of the different PHBV-based blends comprising different PHBV:PCL and PHBV:PLA ratios.

The wetting characteristics of the PHBV-based blends have been investigated by evaluating the water contact angle. To determine whether the film is hydrophobic or hydrophilic, if the angle is between 0° and 90° the surface is considered hydrophilic, being hydrophobic for water contact larger angles. As shown in Figure 3, neat PHBV films are characterized by a hydrophilic behaviour with a contact angle of around 80°, which is in contrast with some reports on the hydrophobic characteristics of PHBV [35], but in line with the observations from Chang and co-workers [36], demonstrating the dependence on the wettability of the materials on processing conditions and morphology. In the case of neat PCL and PLA samples, it can be observed that they are hydrophobic biopolymers, with contact angles of 94° and 96°, respectively [37,38]. Thus, after PCL and PLA addition, PHBV-based blends show an increase in water contact angles, compared to neat PHBV. In the PHBV/PCL blends, the values significantly increased, ranging from 80° (neat PHBV) to 114° (50/50 ratio). Blending PHBV with PLA results in a contact angle value increase from 80° (neat PHBV) to 109° (25/75 ratio) in the PHBV/PLA blends. Thus, blending PHBV with both PCL and PLA results in more hydrophilic surfaces, which is also preferred for anti-fouling surfaces and medical and packaging applications, among others.

### 3.2. Vibrational Spectra

**Figure 4:**
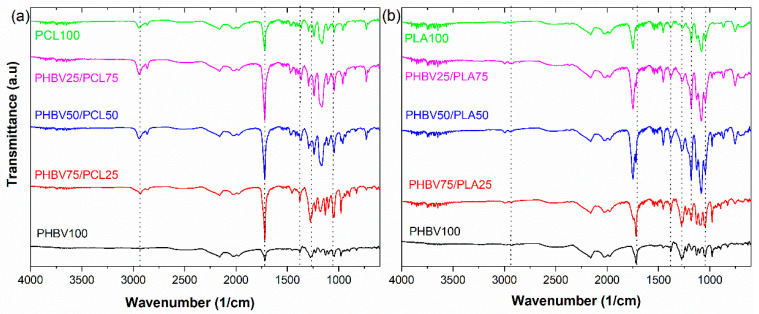
FTIR spectra of the different samples: (**a**) PHBV/PCL and (**b**) PHBV/PLA.

The changes in functional groups of PHBV-based blends were examined through the FTIR spectroscopy technique. Figure 4a shows the spectral variations in the PHBV/PCL blend. The PHBV100 sample presents the characteristic absorption bands of the neat polymer. C=O stretching vibration of PHBV appears at 1720 cm^−1^, while the C-O stretching bands are found at 1278 and 1054 cm^−1^. In addition, the C-H stretching band at 2935 cm^−1^, and the C-H bending vibrations appear at 1453 and 1380 cm^−1^ [39], respectively. Increasing PCL content in the sample leads to a very sharp signal at 1750 cm^−1^, which corresponds to the ester group [40]. Figure 4b shows the spectra of the PHBV/PLA samples, showing that as the presence of PLA increases, small changes are observed in the spectrum. The peak at 1750 cm^−1^, which belongs to the C=O stretching, is clearly visible and hardly varies in the spectra as the amount of PLA increases. In addition, the peak at 1180 cm^−1^, which belongs to the C-O-C stretching, becomes more visible [41]. The vibrational modes at 2848–2997 cm^−1^ and 1371–1458 cm^−1^ are attributed to the stretching and bending vibrations of the C-H groups of the PLA, respectively [42]. Based on the FTIR spectra, it can be concluded that, with the immiscibility of the polymers and the lack of novel bonds and interactions, the polymer component of the blends maintains their main vibrational bands.

### 3.3. Thermal Properties

**Figure 5:**
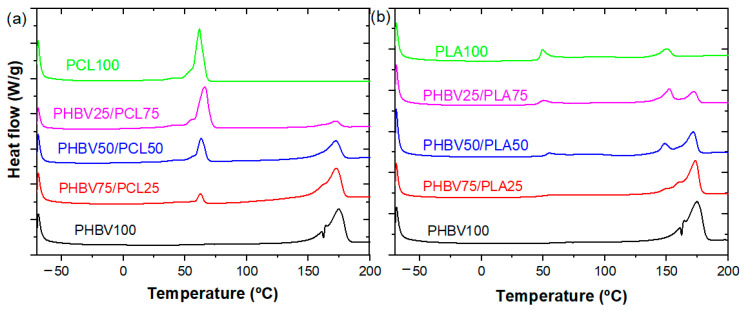
DSC thermograms for the different (**a**) PHBV/PCL and (**b**) PHBV/PLA samples.

The thermal characterization of neat PHBV, PCL, and PLA polymers and PHBV/PCL and PHBV/PLA blends was performed by DSC. Figure 5a shows the DSC thermograms of the different PHBV/PCL blends. The corresponding thermal parameters are summarized in Table 1. Neat PHBV samples show an endothermic peak at 174 °C, which corresponds to its melting point (*T_m_*). As the proportion of PHBV decreases in the blends, this peak disappears and a new endothermic peak appears around 61 °C, which corresponds to the melting temperature of PCL [43]. On the other hand, Figure 5b shows how PHBV follows the same behaviour for the PLA blends, as previously described for the PCL ones, where the endothermic peak at 174 °C disappears as the proportion of PHBV in the mixture decreases. In the same way, an endothermic peak appears more clearly at around 150 °C as the presence of PLA becomes more noticeable. The melting temperature of PHBV is very close to the one of PLA, which facilitates their blending, as demonstrated by the morphological SEM results (Figure 2). Apart from this, around 55 °C, a small glass transition peak corresponding to the glass transition temperature (*T_g_*) is observed for high PLA content [44].

The degree of crystallinity *X_c_* is calculated according to the Equation (5):

(5) $$X_{C}=\frac{\Delta H_{m}}{\Delta H_{m}^{\theta }}\times 100\%$$

where ${\Delta H}_{m}$ is the apparent melting enthalpy of the samples obtained from the integral of the melting peaks, and ${\Delta H}_{m}^{\theta }$ is the melting enthalpy (theoretical) of a 100% crystalline sample. In particular, the ${\Delta H}_{m}^{\theta }$ enthalpy of melting for 100% crystalline samples was 146.6 J·g^−1^ for PHBV [45], 93.7 J·g^−1^ for PLA [46], and 135 J·g^−1^ for PCL [47]. The degree of crystallinity of the samples is given in Table 1. With the addition of PLA or PCL polymers, the crystallinity of PHBV is found to increase at first for the 75/25 blend, and then decrease with the decrease in PHBV content. It indicates that the presence of the blending polymers suppress the crystallization of PHBV, which is attributed to confinement effects, based on the immiscibility of PHBV with PCL and PLA. Further, the presence of both polymers in the blends leads one polymer to act and defect to the crystallization of the other through specific chemical and chemical interactions hindering crystallization [48].

### 3.4. Mechanical Properties

**Figure 6:**
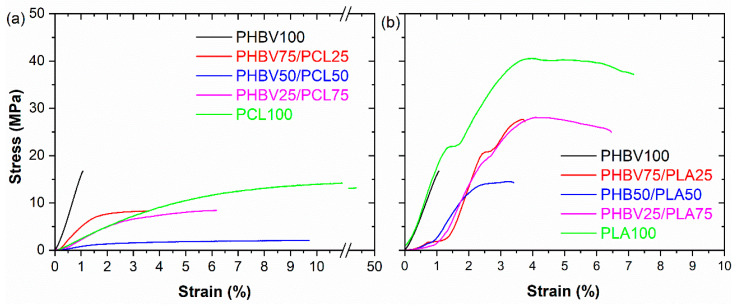
Stress–strain characteristic curves of the (**a**) PHBV/PCL and (**b**) PHBV/PLA blends.

For the analysis of the mechanical properties of the samples, tensile tests were carried out. The stress–strain measurements were used to determine Young's modulus *Y* (slope in the stress–strain curve within the initial linear elastic region) and breaking stress (*ε_b_*) and strain at break (*σ_b_*) of the films. Figure 6a shows the influence of different PHBV/PCL ratios on the mechanical response, while Figure 6b shows the measurement for the PHBV/PLA samples. The corresponding Young's modulus and breaking strain and stress values are presented in Table 2.

Figure 6a shows that neat PHBV is characterized by a low elongation before break and that increasing PCL content leads to an increase in elongation. In fact, the elongation at the break of neat PCL is considerably higher compared to the other samples and it is also remarkable how it stretches plastically before the breaking point [49]. Also, the introduction of PCL leads to a considerable decrease in Young´s modulus from 17 (for PHBV100) to 2 MPa (for PCL100). In particular, it is to notice the strong decrease in the Young modulus for the PHVB50/PCL50 sample, attributed to the mode defective microstructure, as shown by the presence of voids in Figure 2. In addition, Figure 6b shows the effects of PLA on the mechanical response of PHBV-based blends. It is observed that adding PLA results in a significant increase in the breaking strain and stress values [50]. At the same time, the introduction of PLA leads to a general decrease in the Young modulus, being between the 17 MPa of PHBV100 and the 20 MPa for PLA100 for the blend samples. Additionally, compared with PCL, the PLA addition improves the strain at break and the strain at yield, which should be correlated with the SEM results, where the inclusion of PLA leads to a more compact morphology in the PHBV-based blends. In any case, it is noticed that the variation of the mechanical properties is not proportional to the relative blend content, depending on factors like the specific distribution and morphology of the different phases within the blend [51]. Also, significant differences are observed in the values of the Young modulus with respect to related polyester-based polymers and blends [52,53].

### 3.5. Dielectrics Response

**Figure 7:**
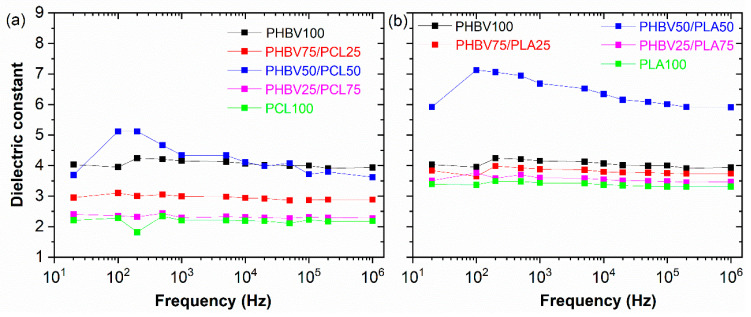
Dielectric constant versus frequency for the (**a**) PHBV/PCL and (**b**) PHBV/PLA samples.

The dielectric spectra for the PHBV/PCL and PHBV/PLA blend with different relative polymer concentrations are shown in Figure 7a,b, respectively. The real part of the dielectric constant *ε*′ shows a slight decrease with increasing frequency, ascribed to slow dipolar dynamics and, therefore, to dipolar relaxation processes with increasing frequency [54]. The values of dielectric constant, loss tangent, and a.c. electrical conductivity at a frequency of 1 kHz are recorded in Table 3. The dielectric constant of the neat PHBV is observed to be *ε*′ *=* 4.16 at 1 kHz. By blending with PCL, the dielectric constant decreases gradually with PCL content, reaching a value of 2.17 at 1 kHz for neat PCL. In addition, the dielectric constant decrease also with increasing PLA content, until a 3.47 at 1 kHz. However, the dielectric constant reaches a maximum value at a 50/50 ratio concentration for the PHBV/PCL and PHBV/PLA blends. Furthermore, the a.c. electrical conductivity of the neat PHBV samples was 5.36 × 10^−11^ S·cm^−1^, which decreases gradually with the addition of different PCL or PLA contents (Table 3). At the same time, the a.c. electrical conductivity, as well as the dielectric losses, reaches a maximum value at a 50/50 ratio concentration for the PHBV/PCL and PHBV/PLA blends. This is explained by the larger interfacial effects at the intermediate concentration, which increases both dielectric response and a.c. conductivity due to the Maxwell–Wagner–Sillars effects [55]. Further, the main factor explaining the variation of the overall electrical response of the samples, together with the interfacial effects, is the large differences in polarity between PHBV [56] and PCL [57] and PLA [58].

### 3.6. Degradability Testing

**Figure 8:**
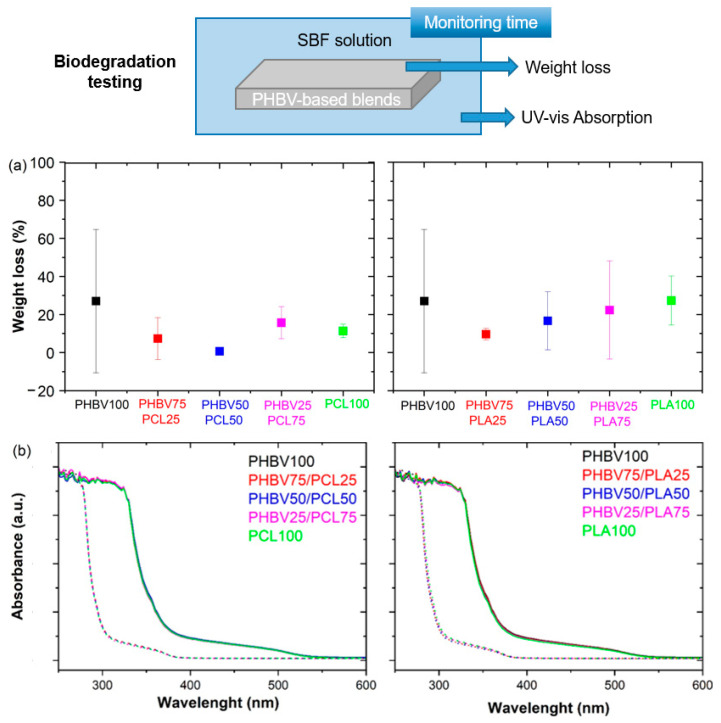
(**a**) Weight loss relative to the original mass after degradation for 8 weeks in SBF solution for the PHBV-based blends. (**b**) UV-Vis absorption spectra before (continuous line) and after degradation for 8 weeks (dashed line) in the obtained SBF solution for the PHBV-based blends.

The biodegradability of the PHBV-based blends was evaluated after immersion in the SBF solution. Specifically, biodegradability was verified by examining the weight loss over a test time period of 8 weeks (Figure 8a). As observed, the weight loss of the samples did not change significantly between samples with different blend ratios and remained within around 20% after the final degradation time testing. This indicates that the physicochemical degradation of neat polymers and blends remains similar, independently of the blend content. Additionally, analysis of UV-vis spectroscopy spectra (Figure 8b) carried out for the SBF solution after the immersion testing, revealed that the curves of absorption spectra of all prepared samples (continuous line) are similar, where a strong and broad absorption band at around 250–400 nm appears. However, the absorption bands shifted to short wavelengths after 8 weeks (dashed line), suggesting the presence of degradation products from the PHBV-based blends in the SBF solution. In summary, these analyses show that blending with PCL and PLA did not significantly change the biodegradation of PHBV-based blends, which is a relevant issue to consider for biomedical applications.

### 3.7. Antibacterial Results

**Figure 9:**
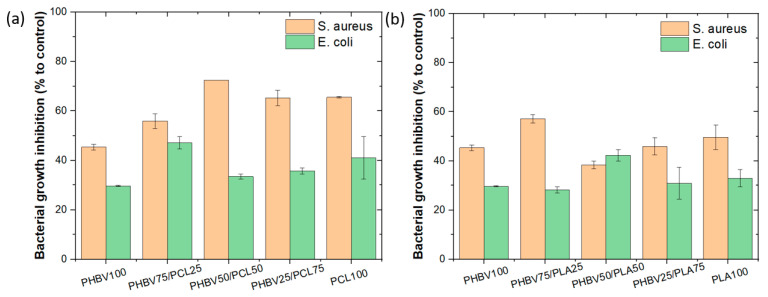
Bacterial growth inhibition of *S. aureus* and *E. coli* with respect to the control of freely growing bacteria for (**a**) PHBV/PCL, and (**b**) PHBV/PLA samples. All results are the average of three independent experiments.

The potential antibacterial activity of the PHBV-based blends was investigated by evaluating their inhibition of bacterial growth. In Figure 9, it is observed the inhibition of growth of *S. aureus* and *E. coli* when in contact for 2 h with the different samples, using the shake flask method. It is observed that all samples are able to inhibit at least 40% of bacteria *S. aureus* growth. However, when it comes to inhibiting *E. coli* growth, all samples performed worse than against *S. aureus*. This fact is related to the structural arrangement of the cellular membrane since Gram-positive *S. aureus* has a tick cell wall and Gram-negative *E. coli* presents a relatively thin cell wall covered by another protective outer membrane [59]. These changes in the cell membrane provide the cell with different characteristics, most notably in reaction to the external environment. Moreover, neat PHBV has an antibacterial inhibition of 45% against *S. aureus* and 30% against *E. coli*. The presence of PCL in the blends (Figure 9a) leads to an increase in bacterial inhibition up to 72% in *S. aureus* (50/50 ratio) and 47% in *E. coli* (75/25 ratio). However, varying PLA content (Figure 9b) leads to no significant differences compared to neat polymers. Regarding the antibacterial mechanism, surface chemistry, wettability, surface charge, roughness, and topographical configuration are the most important factors affecting bacteria behaviour on polymer surfaces [60,61]. The inclusion of PLA and PCL in the PHBV-based blends leads to an increase in the water contact angle (Figure 3), which, together with the morphological variations (Figure 1 and Figure 2) leads to the observed variations in bacterial behaviour. These findings show that the combination of PHBV and PCL leads to promising antibacterial activity, which can be used for biomedical and packaging applications.

## 4. Conclusions

A series of PHBV-based blends with two biodegradable thermoplastics, PCL and PLA with different ratios were successfully produced, and various relevant characteristics for application, such as morphology, mechanical, hydrophilicity/hydrophobicity, degradability, and antibacterial ability were analysed. The results demonstrated that the blending with these polymers affects the morphology, resulting in more compact samples for higher PCL and PLA blending ratios and that the addition of PLA has a more significant effect on all ratios composition. Also, it is shown that the water contact angle is in accordance with the results of morphological tests the blends with PCL and PLA addition exhibit higher contact angles, reducing the surface hydrophilicity. In addition, FTIR results indicate a lack of novel bonds and interactions between PHBV and PCL or PLA. Further, DSC results evidenced that the melting behaviour of PHBV was altered with the addition of PCL and PLA, basically presenting the main features of the different compounds. Regarding the mechanical properties, a large tuning capability is found in terms of both Young’s modulus, ranging between 17 MPa for PHBV to 20.2 MPa for PLA and 2 MPa for PCL, and stress and strain at break. Finally, a remarkable tailorability modification of the dielectric response is observed for the PHBV-based blends, both in terms of dielectric response, ranging between 2.29 for PHBV25:PCL75 and 6.99 for PHBV50:PLA50, and a.c. electrical conductivity, mainly attributed to interfacial effects between polymers. The antibacterial performance of the PHBV is confirmed, which can be improved by the addition of PCL. The results confirm that PHBV blends with PCL and PLA are suitable for the development of sustainable materials for food packaging, sensing, and biomedical applications, among others.

## Figures and Tables

**Table 1:** Thermal properties of the PHBV-based blends.

Sample	*T_m_* (°C) ± 1 °C	*T_m_* (°C) ± 1 °C	*T_g_* (°C) ± 1 °C	*T_g_* (°C) ± 1 °C	*X_c_* (%) ± 2%	*X_c_* (%) ± 2%
	**PHBV**	**PCL**	**PHBV**	**PCL**	**PHBV**	**PCL**
PHBV100	174	-	-	-	41	0
PHBV75/PCL25	172	62	-	-	48	5
PHBV50/PCL50	172	63	-	-	26	19
PHBV25/PCL75	172	66	-	-	8	48
PCL100	-	61	-	-	0	49
	**PHBV**	**PLA**	**PHBV**	**PLA**	**PHBV**	**PLA**
PHBV100	174.	-	-		41	0
PHBV75/PLA25	173	-	-	56	45	0
PHBV50/PLA50	171	148	-	55	16	7
PHBV25/PLA75	172	152	-	50	7	13
PLA100	-	150	-	49	0	12

**Table 2:** Young’s modulus, strain, and stress at break for neat PHBV, PCL, and PLA samples and the corresponding PHBV-based blends.

Sample	*Y* (MPa)	*ε_b_* (%)	*σ_b_* (MPa)
PHBV100	17 ± 0.8	1.7 ± 0.4	16.5 ± 0.5
PHBV75/PCL25	5.7 ± 0.9	4.0 ± 1.9	9.2 ± 0.5
PHBV50/PCL50	0.9 ± 0.1	9.9 ± 0.1	2.1 ± 0.2
PHBV25/PCL75	3.1 ± 0.2	6.2 ± 0.2	8.4 ± 0.2
PCL100	2 ± 0.4	49.3 ± 0.6	13.2 ± 0.5
PHBV100	17 ± 0.8	1.7 ± 0.4	16.5 ± 0.5
PHBV75/PLA25	19.7 ± 1.5	3 ± 0.2	27.3 ± 2.3
PHBV50/PLA50	10 ± 0.7	4.5 ± 0.8	14.3 ± 0.2
PHBV25/PLA75	13.5 ± 1.4	5.2 ± 1.2	24.6 ± 0.7
PLA100	20.2 ± 1.9	6.0 ± 1.6	37.4 ± 3.3

**Table 3:** Dielectric constant, loss tangent, and a.c. electrical conductivity values at 1 kHz for the PHBV-based blends.

Sample	*ε′* at 1 kHz	Loss Tangent at 1 kHz	*σ′* (S·cm^−1^) at 1 kHz
PHBV100	4.16	0.023	5.36 × 10^−11^
PHBV75/PCL25	2.99	0.018	2.92 × 10^−11^
PHBV50/PCL50	4.34	0.233	6.38 × 10^−10^
PHBV25/PCL75	2.29	0.024	3.09 × 10^−11^
PCL100	2.17	0.003	3.05 × 10^−11^
PHBV100	4.16	0.023	5.36 × 10^−11^
PHBV75/PLA25	3.88	0.019	4.22 × 10^−11^
PHBV50/PLA50	6.69	0.059	2.21 × 10^−10^
PHBV25/PLA75	3.59	0.015	2.98 × 10^−11^
PLA100	3.47	0.006	1.08 × 10^−11^

## Data Availability

Data available on request due to restrictions of privacy. The data presented in this study are available on request from the corresponding author. The data are not publicly available due to restrictions of privacy.
